# A comparison of different approaches for imaging cracks in composites by X-ray microtomography

**DOI:** 10.1098/rsta.2016.0037

**Published:** 2016-07-13

**Authors:** B. Yu, R. S. Bradley, C. Soutis, P. J. Withers

**Affiliations:** 1 Henry Moseley X-ray Imaging Facility, School of Materials, University of Manchester, Manchester M13 9PL, UK; 2 Aerospace Research Institute, University of Manchester, Manchester M13 9PL, UK

**Keywords:** four-dimensional imaging, carbon fibre composite, damage evolution, image-based modelling

## Abstract

X-ray computed tomography (CT) has emerged as a key imaging tool in the characterization of materials, allowing three-dimensional visualization of an object non-destructively as well as enabling the monitoring of damage accumulation over time through time-lapse imaging. However, small defects and cracks can be difficult to detect, particularly in composite materials where low-contrast, plate-like geometries of large area can compromise detectability. Here, we investigate a number of strategies aimed at increasing the capability of X-ray CT to detect composite damage such as transverse ply cracking and delamination, looking specifically at a woven glass fibre-reinforced three-dimensional composite. High-resolution region of interest (ROI) scanning, *in situ* loading, phase contrast and contrast agents are examined systematically as strategies for improving the defect detectability. Spatial resolution, contrast, signal-to-noise ratio, full width at half maximum, user friendliness and measurement time are all considered. Taken together, the results suggest that high-resolution ROI scanning combined with the increased contrast resulting from staining give the highest defect detectability.

This article is part of the themed issue ‘Multiscale modelling of the structural integrity of composite materials’.

## 1. Introduction

In recent years, X-ray micro-computed tomography (micro-CT) has emerged as a useful characterization tool in the field of materials, owing to its ability to provide high-resolution three-dimensional images non-destructively [1–3]. Because it provides full three-dimensional morphological information, it can help to delineate the relationship between structure, defects and ultimate failure [4] and can underpin realistic image-based finite-element modelling [5,6].

The use of X-ray tomography in the analysis and identification of damage and microstructures in composites has attracted increasing attention recently [7–10]. First, it provides a non-destructive way of characterizing the sample, without preparation issues which are often associated with scanning electron and optical microscope examinations. Thus, the integrity of the specimen can be preserved. Second, composite materials often fail by the interaction of many damage mechanisms acting across multiple scales in an inherently three-dimensional manner [11], involving matrix cracking, fibre failures, fibre–matrix debonding or delamination. Microvoids, the fibre/matrix interface and fibre orientation also have a strong effect on the damage formation. X-ray CT is superior to the traditional two-dimensional cross-sectional imaging, capturing a three-dimensional view of the complex morphology of microstructures and damage. Finally, as a non-destructive tool, it enables one to track the evolution of various damage mechanisms over time on a single sample. It can be applied either continuously for tracking fast events or using time-lapse methods (e.g. for progressive tensile straining [8] and fatigue [12]).

While X-ray CT offers significant advantages for the imaging and quantification of damage, it also encounters some limitations. The detectability of small features is limited, with a trade-off between the image resolution and sample size that can fit in the field of view (FOV) [13]. This is especially problematic for three-dimensional woven composite materials where the periodic nature of the weave may extend over many millimetres, yet cracking and delamination damage may lead to cracks of submicrometre width. This can be offset to some extent either by using stitching strategies bringing together multiple images [12] or by using region of interest (ROI) approaches [14]. As discussed in Maire & Withers [15], in contrast to point defects such as rounded pores, cracks can often be detected even when they are narrower than a pixel. The narrowest crack opening that can be measured by tomography is primarily dependent on the spatial resolution (normally around two or three pixels) and the contrast difference [1]. However, the presence of cracks of subpixel width can be detected [16]. Further, the detectability of cracks can be improved in a number of ways; here, we consider four strategies:

I. Increasing the resolution: previous work [16] has suggested that a crack can be detected when it is only 10% of a pixel. While this may be optimistic for low-contrast systems, decreasing the pixel size would certainly be expected to increase the detectability of a crack.
II. Imaging cracks under load: when a crack is completely closed, it is undetectable by X-ray tomography. Imaging the composite under load will tend to open the cracks and increase their visibility. Previously, *in situ* loading has been used successfully to highlight the cracks [17].
III. Exploiting phase contrast: it has been recognized for some time [9] that the enhanced edge contrast arising from phase contrast can be used to increase the detectability of cracks. For many laboratory X-ray CT systems, it is not possible to obtain any phase contrast, but here we exploit in-line (propagation-based) phase contrast [18,19] in the Xradia microXCT-400 system by increasing the source to detector distance to examine the efficiency of this approach.
IV. Using a contrast agent: Schilling *et al*. [7] found that in their case adding a contrast agent could improve the detectability of a crack by a factor of 4 from 20% of a pixel to 5% of a pixel. However, it should be remembered that a contrast agent can only illuminate cracks that are interconnected up to the surface, limiting the applicability of this technique. Equally, there are question marks as to the effect of contrast agents on subsequent properties of the composite if used in time-lapse studies [20,21].

Researchers have begun to consider the issues of resolution, sample preparation and length scales by comparing results using different imaging conditions across both laboratory and synchrotron X-ray sources [22,23]. However, to the best of our knowledge, no systematic study has been carried out to evaluate the effectiveness of different strategies for increasing the detectability of cracks and damage features. In this paper, we look at the effects of spatial resolution, phase contrast, staining and applied loading, for detecting damage in a three-dimensional angle-interlocked (AI) woven glass fibre-reinforced composite when using a laboratory X-ray CT scanner. The effectiveness of each of the above-mentioned strategies for improving defect detection either in isolation or in combination is investigated with the aim of providing some guiding principles.

## 2. Material and experimental methods

### (a) Fabrication of three-dimensional woven composite specimens

**Figure 1.**
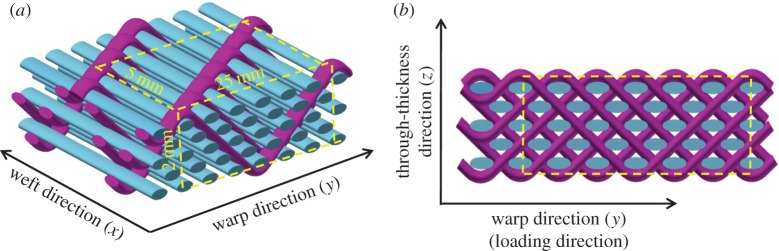
Idealized schematics show the angle-interlocked woven fabric (*a*) and its cross-section (*b*). Weft, binder and additional warp yarns are coloured blue, purple and pink, respectively. (Adapted from [24].)

The three-dimensional AI woven preform was fabricated as a single integral structure, using a conventional rapier weaving machine. The fibre architecture is illustrated in figure 1, which shows that the five layers of weft yarns (blue) are held together by the binder yarns (purple) through the whole thickness of the fabric. The unit cell of this three-dimensional preform, with dimensions of 25×5×2 mm, is outlined in the yellow rectangle in figure 1. It is worth noting that all the yarns identically consist of S-2 high-strength glass fibres. Further details about the three-dimensional woven preform can be found in our previous study [24].

**Figure 2.**
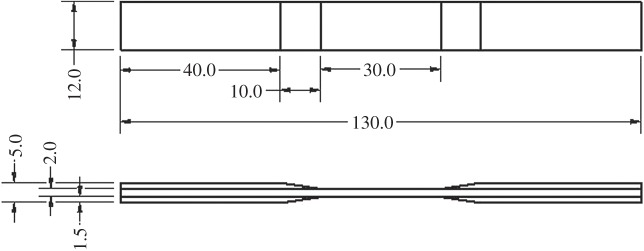
Sample test-piece geometry (dimensions in mm).

To fabricate the three-dimensional composite panel, the three-dimensional fabric was infused with epoxy resin in a sealed bag using the vacuum resin infusion process as described in detail elsewhere [24]. In order to ensure that the damage occurred within the FOV of the CT system, sample test pieces were smaller than the standard ASTM design [25]. The sample width and gauge length were 12 and 30 mm, respectively, ensuring at least two unit cells across the width of the test coupon and one along its length (figure 2); 50 mm long glass fibre/epoxy end tabs were glued onto two sides of the composite plate, and all the sample test pieces were subsequently cut out in the warp direction.

### (b) Static tensile and fatigue testing

Static tensile testing was performed on a set of five AI samples along the warp direction to verify whether these small samples display the properties representative of the composite panel obtained through standard-sized test pieces (table 1). These results clearly show that smaller composite samples exhibit similar properties to standard-sized ones.

**Table 1.** The comparison of the tensile properties of the small and standard sample test pieces.

	small sample test piece	standard sample test piece
gauge size (width×length in mm^2^)	12×30	20×100
ultimate tensile strength (MPa)	462±20	467±35
ultimate tensile strain (%)	3.66±0.15	4.01±0.37
Young's modulus (GPa)	17.4±1.5	17.7±0.5

Tension–tension fatigue loading was conducted using a sinusoidal waveform, with a minimum-to-maximum stress ratio *R*=0.1, at a frequency of 5 Hz. The maximum stress applied in the testing is 210 MPa, corresponding to 45% of the mean ultimate tensile strength (UTS). A fatigue test was only regarded as valid when ultimate failure occurred within the gauge. The results of three valid tests are shown in table 2.

**Table 2.** Cycles to failure of small sample test pieces recorded in valid tension–tension fatigue tests at *R*=0.1 and a maximum stress of 210 MPa (45% UTS).

sample number	fatigue life, *N*_f_ (cycles)
I	72 061
II	111 640
III	84 419
average	89 373

In order to assess the extent to which different damage mechanisms can be detected by the different delineation strategies, the fatigue damage was assessed in a sample that had experienced 50 000 fatigue cycles with a maximum stress level of 45% UTS. This represents approximately 55% of the total fatigue life. Previous work on three-dimensional woven composites [12] found that by this stage all the possible types of fatigue damage that arise in this material are evident except for longitudinal fibre fracture, which only appeared just before final failure.

### (c) Computed tomography scans

A systematic series of scans were conducted on the fatigued (approx. 55% of life) AI sample using ROI, staining, phase contrast and *in situ* loading in turn as ways to better identify the presence of defects. For a direct comparison, each scan was focused on approximately the same location. The CT scans were undertaken using a Zeiss Xradia Micro 400 XCT system, which is equipped with multiple magnification lens options. A source voltage of 100 kV and current of 100 μA were used in all cases. The radiographs were collected on the 2k×2k, 16-bit high-resolution cooled CCD detector.

In order to make fair comparisons between the different strategies as the different parameters (table 3) are varied, we have endeavoured to maintain as many aspects constant as possible. For example, when using the same optical lens, it is important to keep the recorded signal approximately constant in each projection, because increasing the number of detected photons will give better counting statistics and thereby affect the signal-to-noise ratio (SNR). However, in order to control the level of phase contrast, the distance between source to sample, *R*_1_, and sample to detector, *R*_2_, may vary. Therefore, the exposure time must be changed accordingly to ensure that approximately the same number of photons is acquired in each case. Because the number of counts is inversely proportional to the square of the distance between the source and the detector, (*R*_1_+*R*_2_)^2^, the exposure time required can be calculated from equation (2.1) with varying *R*_1_ and *R*_2_
2.1
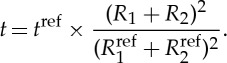
Taking scan 1 in table 3 as the reference scan (i.e. moderate (8.9 μm) spatial resolution, no load, no phase contrast and no contrast agent), which gives the standard time and sample to source and detector parameters 
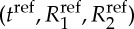
, the exposure time required for the other images can be determined.

**Table 3.** A summary of the key scan settings. FOV represents the width of a single image (mm).

	mag. (×)	phase contrast	*in situ* loading	contrast agent	projection (s)	scan time (h)	no. projections	*R*_1_ (mm)	*R*_2_ (mm)	pixel size (*μ*m) (and FOV (mm))
scan 1	1	×	×	×	10.5	3	1001	35	18.5	8.9 (10.4)
scan 2	1	×	√	×	65	18	1001	85	45	8.9 (10.4)
scan 3	1	×	×	√	10.5	3	1001	35	18.5	8.9 (10.4)
scan 4	4	×	×	×	10.5	3	1001	35	18.5	2.2 (4.4)
scan 5	4	×	√	×	65	18	1001	85	45	2.2 (4.4)
scan 6	4	×	×	√	10.5	3	1001	35	18.5	2.2 (4.4)
scan 7	4	√	×	×	140	39	1001	127.5	67.5	2.2 (4.4)
scan 8	4	√	×	×	140	39	1001	170	90	2.2 (4.4)
scan 9	4	√	×	√	140	39	1001	127.5	67.5	2.2 (4.4)
scan 10	4	√	×	√	140	39	1001	170	90	2.2 (4.4)

The *R*_1_-to-*R*_2_ ratio must be held constant for a given optical magnification to ensure the same geometrical magnification and thereby the same effective pixel size. It is clear from table 3 that the time per scan varies enormously (by more than a factor of 10) when applying the different scan settings. Table 3 may also influence one's choice of the most appropriate settings. Given that the width of the sample imaged in the FOV varies with different magnifications, the time to tessellate a number of images to build up an image of the same area varies even more steeply.

After acquiring all the scans of the sample in the unstained state (scans 1, 2, 4, 5, 7 and 8), the sample was then stained to explore the extent to which staining is able to delineate those cracks that are not visible under strategies I–III. The dye penetrant and staining procedures are as described in [24]. In essence, the sample was soaked in a dye penetrant solution comprising 250 g zinc iodide, 80 ml distilled water, 80 ml isopropyl alcohol and 1 ml Kodak photoflow at room temperature for 24 h. Note that the excess dye on the sample surface was wiped off prior to scanning.

**Figure 3.**
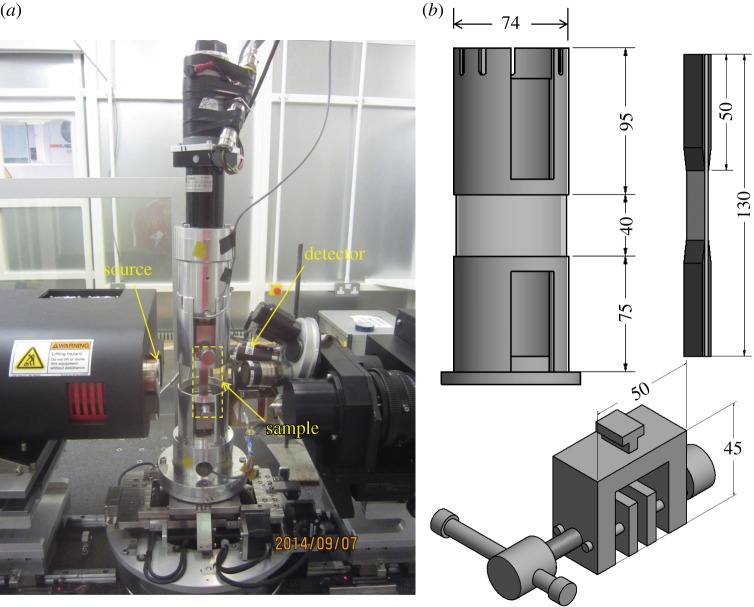
(*a*) The scan set-up, showing that the loading rig was situated at the minimum distance to the detector and source. (*b*) Illustration of the different parts of the loading rig, including the modified tube, composite sample and the miniaturized screw side action grip.

In order to accommodate the *in situ* loading stage and to avoid it striking the source or detector while allowing a full 180° rotation, *R*_1_ and *R*_2_ were maintained at a safe distance as shown in figure 3. During scans 2 and 5, approximately 1400 N was applied to the sample to increase the crack opening: this corresponds to just 25% of the maximum loading during the fatigue testing. Visual inspection of radiographs indicated that this load was sufficient to open the cracks. The displacement control mode of the loading rig was used to avoid any damage to the sample during the long periods required for data acquisition.

Despite the fact that the plastic tube (figure 3) has very low X-ray absorption, the composite is also lowly attenuating with the result that the plastic tube affects the overall attenuation. To account for this, X-ray radiographs were taken of the sample within the rig to calculate an exposure time that delivers approximately the same number of photons to the detector as when the rig (tube) is not in place.

**Figure 4.**
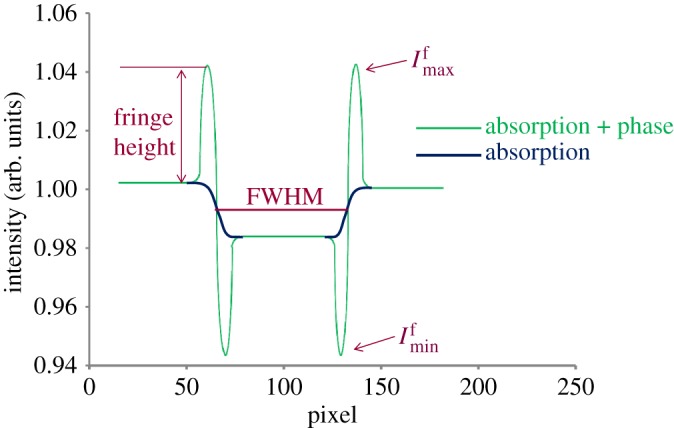
Schematic of the absorption contrast profile and the mixed phase and absorption contrast profile across a crack. (Adapted from [19].)

Under strategy III, *R*_1_ and *R*_2_ were increased in balance to increase the phase contrast [19], whereas the exposure time, *t*, was adjusted accordingly to maintain a similar number of counts, as summarized in table 3. In practice, very large propagation distances (*R*_2_) should be avoided: first, because excessive phase contrast can blur the edge contrast and, second, because increasing the source to detector distance requires much longer acquisition times. The change of phase contrast as a function of *R*_1_ and *R*_2_ was assessed by plotting line profiles across a certain crack on each projection. Referring to [19], the variation in the amount of phase contrast can be quantified by
2.2
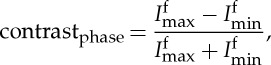
where 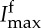 and 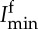 are the maximum intensity of the bright fringe and the minimum intensity of the dark fringe, respectively, as indicated in figure 4. It should be noted from figure 4 that the cracks would not be distinguishable with absorption contrast alone when the attenuation coefficients of cracks and the surrounding background are very similar. Thus, phase contrast is often complementary to the absorption contrast.

**Figure 5.**
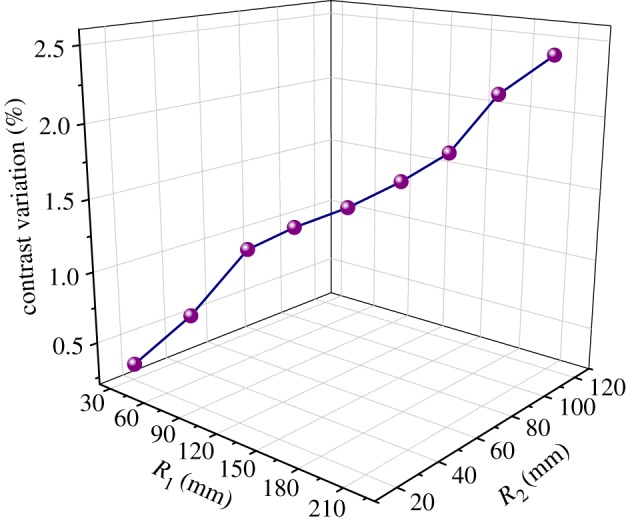
The increase in the phase contrast (contrast_phase_×100%) of a crack recorded in the radiographs with increasing *R*_1_ and *R*_2_.

As plotted in figure 5, the contrast was found generally to increase with increasing *R*_1_ and *R*_2_, although only by a small amount, because the view of the edge of the crack was obstructed by the overlapping features on the projection (radiograph). The line chart has not reached a plateau by the point where *R*_1_=212.5 and *R*_2_=112.5, suggesting that better contrast could be achieved were the source and detector distance increased further. On the other hand, beyond *R*_2_=170, *R*_2_=90, the acquisition time becomes infeasibly long according to equation (2.1). Thus, in this case, we used the same exposure time in scan 7 and scan 8, while setting the longer source to detector distance in scan 8, with the result that only half the photons were collected compared with scan 7. This was done to determine whether the improvement in contrast outweighs the increase in noise without increasing the time per projection to maintain the same illumination.

### (d) Evaluation of reconstructed three-dimensional images

Scans were reconstructed using a standard filtered back projection algorithm in the Xradia XMReconstructor software. The reconstructed volume, which had an intensity range from 0 to 65 535 (16-bit), was visualized using Avizo 9. No pixel smoothing, filtering or greyscale redistribution was applied to any of the volumes, so that direct comparisons can be made.

Depending on the mechanism of contrast formation, a different equation should be used to quantify the contrast of the crack. For the CT scans where phase contrast mainly contributed to the contrast of the crack, it was calculated by equation (2.2). For the CT scans where the contrast of the crack predominantly arose from attenuation, the contrast is defined as the difference in greyscale intensity of the crack and its background, as expressed in
2.3
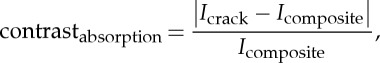
where *I*_composite_ is the mean value of the composite surrounding the crack, whereas *I*_crack_ is calculated by taking an average over 10 individual points located within the crack.

The SNR can be calculated according to
2.4
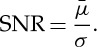
In a homogeneous area of composite containing *m* pixels, each pixel has some value, *μ*_*i*_. 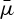 is the mean value of the set of *m* pixels and *σ* is the image noise specified as the standard deviation.

Apart from the image contrast and SNR, the full width at half maximum (FWHM) was calculated. This was done to quantify how the measurement of crack opening can be affected by different techniques. The line profile of each crack was fitted by a Gaussian function, in order to determine the FWHM values.

### (e) Post-mortem sectioning and scanning electron microscopy analysis

Once the non-destructive CT scanning was complete, the sample was sectioned and then imaged in a Quanta 650 scanning electron microscope. Subsequently, the registration module in Avizo was used to register the two-dimensional destructive slice with the three-dimensional datasets by searching for the virtual slice which has the best similarity. Because of the limited amount of information in the slice thickness direction and surface flatness, it is not possible to register the destructive scanning electron microscopy (SEM) cross-sectional view precisely with the corresponding virtual cross sections from the CT images, but best efforts were made to achieve a match.

## 3. Results and discussion

**Figure 6.**
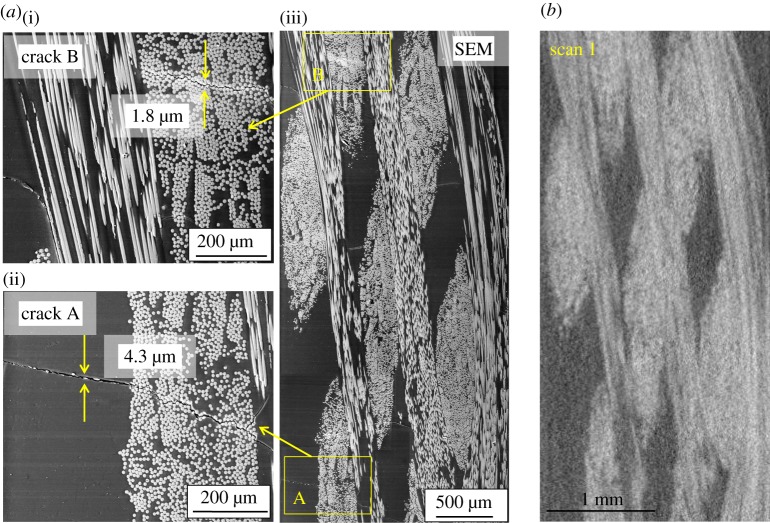
A direct comparison between a post-mortem high-resolutionSEM cross-sectional image (*a*(iii)) with the corresponding virtual slice (*b*) reconstructed from scan 1. The regions of the SEM image containing cracks A and B are magnified (*a*(i)(ii)) to show the crack openings and their morphology.

The cross-sectional SEM micrograph and the corresponding virtual CT slice from scan 1 (the reference tomograph) are compared in figure 6. The openings of two cracks (labelled A and B) shown in the magnified inserts of figure 6 have been estimated by SEM images to be 4.3 and 1.8 μm, respectively. As summarized in table 3, scan 1 was acquired at a moderate pixel size (approx. 9 μm) with a FOV (10.4 mm) sufficient to image most of the sample width. Although the resolution is sufficient to delineate the tow architecture this has resulted in a rather diffuse image, with low resolution of details. By contrast, in the high-resolution SEM image, fine features such as individual fibres, resin pockets and microcracks not apparent in scan 1 appear very prominently. In order to make visual comparisons between volumes recorded using the different scanning strategies the ROIs containing the two cracks labelled in figure 6*a* as ‘crack A’ and ‘crack B’ will be focused on in the rest of the paper along with line profiles of greyscale intensity.

### (a) Region of interest comparisons according to scan strategy

For each of the scans, the ROI containing cracks A and B has been located for side-by-side comparison of the different strategies. Because of the difficulty of exactly matching the slices acquired at different times because of removing and reloading the samples, small differences between the precise locations are unavoidable when correlated manually.

**Figure 7.**
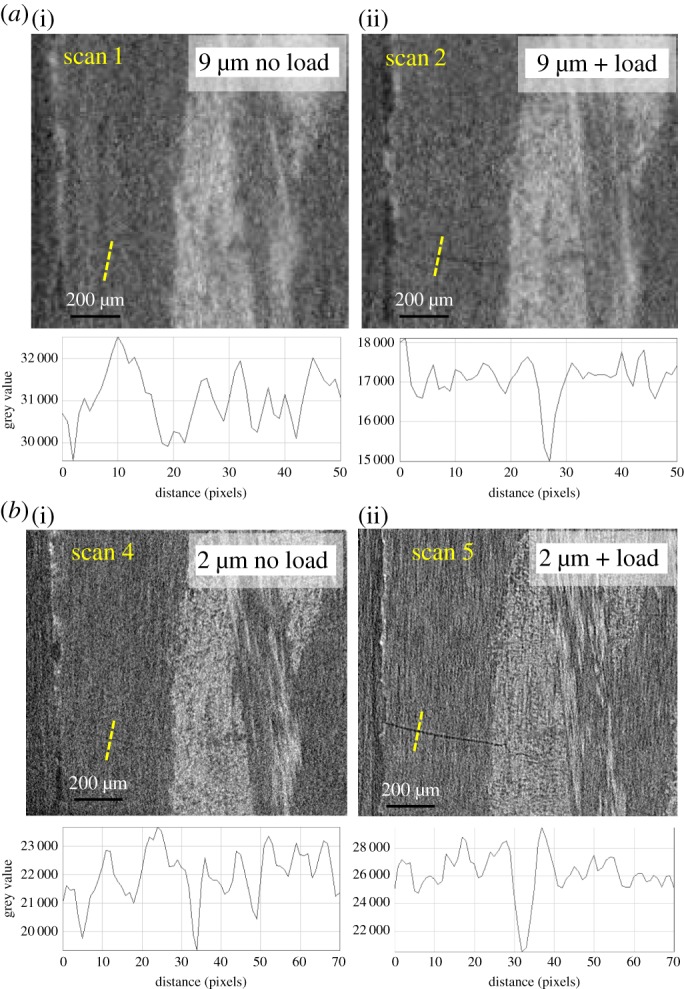
Enlarged virtual CT sections showing the ROI containing crack A. Examining strategies I (increasing the magnification) ((*a*) versus (*b*)) and II (applying a crack opening load) ((*a*(i),*b*(i)) versus (*a*(ii),*b*(ii))). A greyscaleline profile across the location of the crack is shown below each image.

Strategy I. Decreasing the pixel size (increasing the magnification). Figure 7 clearly shows that features which are undetectable at 9 μm pixel size (scan 1, no load) can be observed at 4×higher magnification (scan 4, no load). At the higher magnification, the image is much sharper, clearly delineating the boundary of the weft yarn and the location of some resin-rich regions. However, it is still difficult to visually distinguish the individual fibres or cracks, although a few transverse cracks are visible by naked eye especially in the fibre-free region.

Strategy II. Imaging under a crack-opening load. It is evident by comparing figure 7*a*(i),*b*(i) with figure 7*a*(ii),*b*(ii) that, under modest tensile loading, the cracks can be held open to such an extent that the transverse cracks are more apparent, even at the lower magnification (figure 7*a*(ii)). The line profiles also show evidence of significant crack opening under the applied load. Of course, while this method is well suited to the delineation of transverse cracking, it is not very sensitive to longitudinal splitting or debonding cracks, as the loading is applied in the longitudinal direction and so induces little crack opening for these types of cracks.

**Figure 8.**
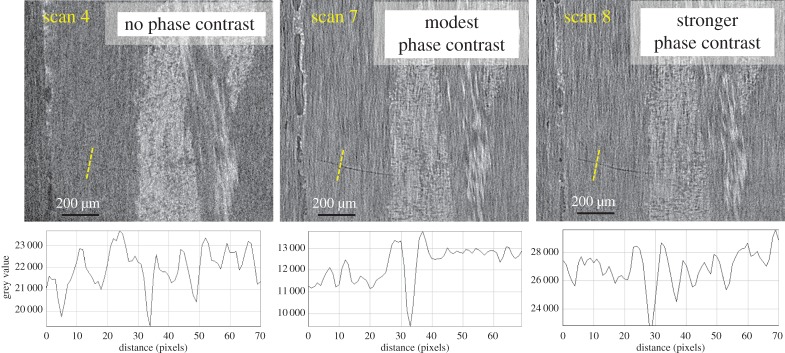
Enlarged virtual sections show the ROI containing crack A. Examining strategy III (increasing the phase contrast from left to right) at 2 μm pixel size. A greyscale line profile across the location of the crack is shown below each image.

Strategy III. Exploiting phase contrast. The effect of increasing levels of phase contrast is investigated in figure 8. This has resulted in sharper edges to the crack than for the attenuation-only image (scan 4). As indicated in table 3, the source to detector distances in scans 7 and 8 were nearly four times and five times, respectively, that in scan 4. This has led to the appearance of phase contrast fringes in the line profiles for scans 7 and 8. It is apparent from the line profiles that for scan 4 the crack is evidenced only by a trough in intensity, whereas in scan 8 it shows two strong intensity peaks on either side of the trough. Although this contrast is not sufficient to easily segment the crack, it does improve the visibility of the crack. Compared with the resin-rich region, the relatively poor detectability of cracks in the fibre-rich region is also worth noting. In fact, crack A has cut through the entire transverse yarns (shown in the SEM observation in figure 6). However, none of the three scans has shown any evidence of it in the region of the transverse fibres because of the more uneven background contrast against which to detect the crack.

**Figure 9.**
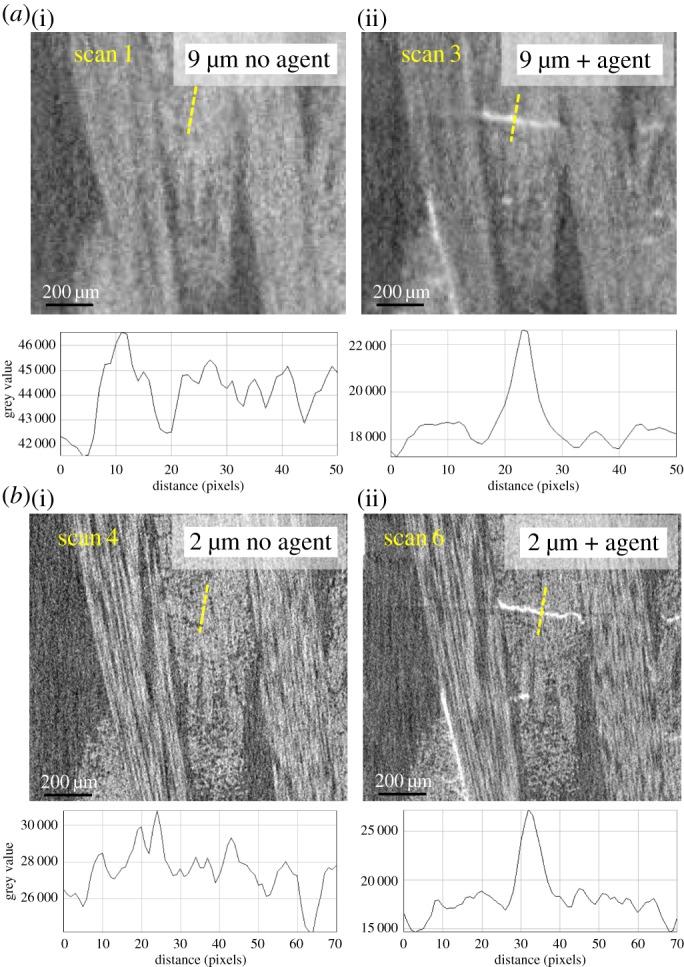
Enlargedvirtual sections showing the ROI containing crack B. Examining strategy IV without (*a*(i),*b*(i)) and with (*a*(ii),(*b*(ii)) contrast agent at both moderate (*a*) and high (*b*) resolution. A greyscale line profile across the same location of the crack is shown below each image.

Strategy IV. Contrast agent. The effects of using contrast agent at both moderate magnification (9 μm pixel size) and high magnification (2 μm pixel size) are shown in figure 9. The distinctive heavy element contrast is clearly observed at both magnifications. Given that at 1×magnification (9 μm pixels) the FOV (10.4 mm) essentially covers the whole gauge, one can expect with staining it can yield reasonably clear imaging of the overall larger-scale damage in the sample. However, owing to the lower image resolution in scan 3, the morphology of the cracks tends to lose some details relative to that in scan 6. Another significant difference between the ROI in figure 9 and those in figure 7 and figure 8 is that the longitudinally oriented defects are clearly resolved.

**Figure 10.**
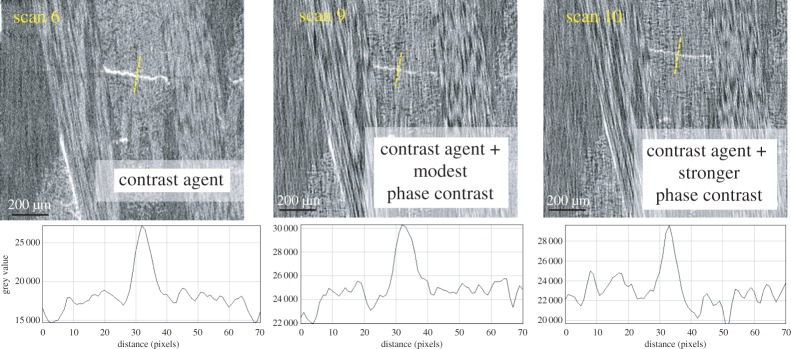
Examination of the effect of combining phase contrast and contrast agent. A greyscale line profile across the location of the crack is shown below each image.

Combining phase contrast and contrast agents (figure 10). Comparison of the ROI containing crack B suggests that combining the use of contrast agent with phase contrast is not particularly helpful from a crack detection viewpoint. Unsurprisingly, with phase contrast the individual fibres are much more pronounced than scan 6 because of the enhanced contrast at the interface between fibre and matrix. However, the contrast of the crack is actually somewhat lower than that without phase contrast. It is perhaps not surprising that there is no synergy between the two mechanisms given that one is related to the real part of the refractive index and one to the imaginary part.

### (b) Contrast and signal-to-noise ratio

In this section, the image quality of the CT slices is quantitatively assessed in terms of the contrast of cracks A and B and SNR in each image.

The results summarized in table 4 confirm that using contrast agent, opening the cracks, increasing the resolution and increasing phase contrast all improve the detectability of transverse cracks. Staining is by far the most effective means of enhancing the contrast of the cracks (scan 6). The influence of image resolution is also marked, because the scans at 9 μm pixel size all show lower contrast than those for 2 μm. This can be explained by the partial volume [26] and smearing effect. When the crack is of the order of, or smaller than, the spatial resolution, the difference in greyscale intensity between the crack and its surroundings tends to become smaller. The results also indicate that holding the crack open with *in situ* loading is an effective way to increase the contrast (scan 5). This is probably because increasing the width of the crack also decreases the smearing effect of the resolution function. The exploitation of phase contrast in scan 7 and scan 8 has boasted the contrast somewhat, although the extent of phase contrast that can be introduced using laboratory instruments is currently limited. Perhaps counterintuitively, the contrast is lower in scans 9 and 10, where phase contrast and staining have both been applied, than in scan 6, where the contrast was only affected by contrast agent.

**Table 4.** Contrast of cracks A and B for the different scans (the values of scans 7 and 8 were calculated by equation (2.2) for phase contrast and the rest by equation (2.3) for absorption contrast).

	scan 1	scan 2	scan 3	scan 4	scan 5	scan 6	scan 7	scan 8	scan 9	scan 10
crack A	zero	0.19		0.20	0.47		0.15	0.14		
crack B	zero		0.31	undetected		0.65			0.37	0.45

The SNR for each CT slice is presented in table 5. It is worth noting that here the SNR values were determined on the same resin-rich region in each slice, as an indication of overall scan quality. It is apparent that scans 1, 2 and 3 give higher SNRs than the other scans. This is mainly owing to the larger pixel size at low magnification, leading to larger sampling size. Interestingly, scans 8 and 10 have a relatively high SNR despite the fact that their signal is considerably less than that of the other scans taken at the same magnification.

**Table 5.** SNR of CT slices recorded by different scans.

	scan 1	scan 2	scan 3	scan 4	scan 5	scan 6	scan 7	scan 8	scan 9	scan 10
SNR	14.8	13.3	17.6	10.1	7.8	9.1	8.4	9.1	9.4	9.7

### (c) Crack opening

In this section, the crack openings as quantified by the FWHM (figure 4) values are compared with the measurements obtained from the high-resolution SEM images for cracks A and B in table 6. The actual crack opening in the absence of load, as measured by SEM, of crack A (4.3 μm) is twice the voxel size in the ROI scan, whereas the width of crack B is 1.8 μm, which is smaller than a pixel. This corroborates two findings. First, crack B is not distinguishable in a normal FOV scan, as it is too narrow compared with pixel size (one-fifth of a pixel). Second, owing to low contrast, the smallest crack that can be detected is generally two to three times the pixel size (e.g. crack A in scan 4), which corresponds to the size of the spatial resolution. Nevertheless, after applying the contrast agent to enhance the contrast, we can detect a crack of less than 1/10th the spatial resolution (one-fifth of a pixel), e.g. crack B in scan 3. However, the crack is broadened to a width of four pixels owing to the partial volume effect, which leads to an averaging of attenuation coefficients. Although the informed use of the partial volume effect allows the subvoxel features to be detected, it will inevitably overestimate the crack openings. All these findings suggest that while CT can detect cracks at or below the spatial resolution the crack openings cannot be measured accurately.

**Table 6.** Full width at half maximum of line profiles (in *μ*m) of the cracks in CT slices recorded by different scans, compared with the crack opening measurements obtained from the high-resolution SEM micrograph. Note that some data are missing in the table: the contrast of crack A was not measured in the cases where the contrast agent did not infiltrate it; the contrast of crack B was not measured in the cases where it was undetected.

		scan 1	scan 2	scan 3	scan 4	scan 5	scan 6	scan 7	scan 8	scan 9	scan 10
	s.e.m.	8.9 μm pixel size			2.2 μm pixel size						
crack A	4.3	undetected	21		7	10		7	8		
crack B	1.8	undetected		42	undetected		13			15	13

## 4. Conclusion

In this study, a series of CT scans were carried out to explore an optimum approach to imaging the fatigue damage in a glass fibre three-dimensional woven AI sample. While maintaining approximately the same level of illumination, four different strategies were applied both singly and in combination to enhance the detectability of cracks, namely: I, improved spatial resolution; II, modest *in situ* loading (25% the fatigue load); III, phase contrast; and IV, contrast agents. All were applied to the same composite sample and, in each case, the same damaged ROIs were examined. Assessment included both the visual detectability of the cracks as well as quantitative measures such as the contrast, SNR and FWHM.

In order to resolve a crack, a pixel must show a sufficient difference in grey level from its neighbourhood, so that it can be distinguished above the noise. This is a combination of both the contrast of the feature and its width. In polymer matrix composite materials, the contrast between a crack and the resin can be small. In all cases, it was possible to detect cracks with a dimension around the spatial resolution (a few pixels). This is significantly less than the 1/10th of a pixel detection limit given by [16], mainly owing to the low contrast. Both increasing the resolution and using a modest applied load to open the cracks were successful in increasing the detectability of the cracks because they essentially increased the size of the crack relative to the pixel size. Phase contrast and contrast agents were also effective because they increase the contrast difference between the crack and the composite. Of all the methods staining was perhaps the most effective, increasing the sensitivity of cracks to better than 1/10th the spatial resolution. Unsurprisingly, the FWHM of crack features is not a good indicator of the actual crack width when they are narrower than the spatial resolution.

Of course different techniques have their limitations; for example, contrast agents can only illuminate surface breaking defects, the use of a crack opening load can only open transverse cracks, increasing the phase contrast gives rise to longer acquisition times when using a laboratory system while increasing the magnification decreases the FOV necessitating ROI scanning of small regions or stitching methods to image larger ones.

From the segmentation point of view, the detectability of cracks can be improved by both phase contrast (illuminating the cracks edges) and *in situ* loading (increasing the crack size). These two strategies can definitely improve the segmentation of cracks to some extent, but are not effective enough to enable all the cracks to be fully segmented. By contrast, the heavy element contrast agent provides significantly higher intensity over the background, capturing more damage modes, such as cracks in the fibre-rich region and longitudinal debonding cracks. Therefore, it is highly likely to achieve an automatic segmentation of the cracks in scan 6, which gives reasonable three-dimensional representation of damage mechanisms.

Overall, we found that a combination of high spatial resolution along with the use of a contrast agent gave the best results, revealing the fatigue damage at microscopic scale. Compared with other techniques, this approach offers good feasibility of scan set-up, providing efficient routine characterization of damage.

## References

[1] Stock SR 1999 X-ray microtomography of materials. *Int. Mater. Rev*.** 44, 141–164. (10.1179/095066099101528261)

[2] Salvo L *et al.* 2003 X-ray micro-tomography an attractive characterisation technique in materials science. *Nucl. Instrum. Methods B* 200, 273–286. (10.1016/S0168-583X(02)01689-0)

[3] Landis EN, Keane DT 2010 X-ray microtomography. *Mater. Charact*.** 61, 1305–1316. (10.1016/j.matchar.2010.09.012)

[4] Withers PJ, Preuss M 2012 Fatigue and damage in structural materials studied by X-ray tomography. *Annu. Rev. Mater. Res*.** 42, 81–103. (10.1146/annurev-matsci-070511-155111)

[5] Ali J, Farooqi JK, Buckthorpe D, Cheyne A, Mummery P 2009 Comparative study of predictive FE methods for mechanical properties of nuclear composites. *J. Nucl. Mater*.** 383, 247–253. (10.1016/j.jnucmat.2008.09.020)

[6] Naouar N, Vidal-Salle E, Schneider J, Maire E, Boisse P 2015 3D composite reinforcement meso FE analyses based on X-ray computed tomography. *Compos. Struct*.** 132, 1094–1104. (10.1016/j.compstruct.2015.07.005)

[7] Schilling PJ, Karedla BR, Tatiparthi AK, Verges MA, Herrington PD 2005 X-ray computed microtomography of internal damage in fiber reinforced polymer matrix composites. *Compos. Sci. Technol*.** 65, 2071–2078. (10.1016/j.compscitech.2005.05.014)

[8] Scott AE, Mavrogordato M, Wright P, Sinclair I, Spearing SM 2011 *In situ* fibre fracture measurement in carbon–epoxy laminates using high resolution computed tomography. *Compos. Sci. Technol*.** 71, 1471–1477. (10.1016/j.compscitech.2011.06.004)

[9] Wright P, Fu X, Sinclair I, Spearing SM 2008 Ultra high resolution computed tomography of damage in notched carbon fiber–epoxy composites. *J. Compos. Mater*.** 42, 1993–2002. (10.1177/0021998308092211)

[10] Lambert J, Chambers A, Sinclair I, Spearing S 2012 3D damage characterisation and the role of voids in the fatigue of wind turbine blade materials. *Compos. Sci. Technol*.** 72, 337–343. (10.1016/j.compscitech.2011.11.023)

[11] Stinchcomb WW 1986 Nondestructive evaluation of damage accumulation processes in composite laminates. *Compos. Sci. Technol*.** 25, 103–118. (10.1016/0266-3538(86)90037-0)

[12] Yu B, Blanc R, Soutis C, Withers PJ 2016 Evolution of damage during the fatigue of 3D woven glass-fibre reinforced composites subjected to tension-tension loading observed by time-lapse X-ray tomography. *Compos. A, Appl. S*. 82, 279–290. (10.1016/j.compositesa.2015.09.001)

[13] Cox BN, Spearing SM, Mumm DR 2008 Practical challenges in formulating virtual tests for structural composites. In *Mechanical response of composites* (eds PP Camanho, CG Dávila, ST Pinho, JJC Remmers), pp. 57–75. Dordrecht, The Netherlands: Springer.

[14] Kyrieleis A, Titarenko V, Ibison M, Connolley T, Withers PJ 2011 Region-of-interest tomography using filtered backprojection: assessing the practical limits. *J. Microsc*. 241, 69–82. (10.1111/j.1365-2818.2010.03408.x) 21118206

[15] Maire E, Withers PJ 2014 Quantitative X-ray tomography. *Int. Mater. Rev*.** 59, 1–43. (10.1179/1743280413Y.0000000023)

[16] Breunig TM, Stock SR, Guvenilir A, Elliott JC, Anderson P, Davis GR 1993 Damage in aligned-fibre SiC/Al quantified using a laboratory X-ray tomographic microscope. *Composites* 24, 209–213. (10.1016/0010-4361(93)90165-5)

[17] Cosmi F, Bernasconi A 2013 Micro-CT investigation on fatigue damage evolution in short fibre reinforced polymers. *Compos. Sci. Technol*.** 79, 70–76. (10.1016/j.compscitech.2013.02.008)

[18] Mayo SC, Stevenson AW, Wilkins SW 2012 In-line phase-contrast X-ray imaging and tomography for materials science. *Materials* 5, 937–965. (10.3390/ma5050937) 28817018 PMC5458972

[19] Bradley RS, McNeil A, Withers PJ 2010 An examination of phase retrieval algorithms as applied to phase contrast tomography using laboratory sources. In *SPIE Optical Engineering+ Applications*, pp. 780 404–780 410. Bellingham, WA: International Society for Optics and Photonics.

[20] Furrow APC, Dillard DA, Clair TLS, Hinkley J 1998 Dye penetrant induced micro cracking in high performance thermoplastic polyimide composites. *J. Compos. Mater*.** 32, 31–48.

[21] Spearing SM, Beaumont PW 1992 Fatigue damage mechanics of composite materials. I: Experimental measurement of damage and post-fatigue properties. *Compos. Sci. Technol*.** 44, 159–168. (10.1016/0266-3538(92)90109-G)

[22] Kastner J, Harrer B, Requena G, Brunke O 2010 A comparative study of high resolution cone beam X-ray tomography and synchrotron tomography applied to Fe-and Al-alloys. *NDT E Int.* 43, 599–605. (10.1016/j.ndteint.2010.06.004) 20976283 PMC2947926

[23] Bull DJ, Helfen L, Sinclair I, Spearing SM, Baumbach T 2013 A comparison of multi-scale 3D X-ray tomographic inspection techniques for assessing carbon fibre composite impact damage. *Compos. Sci. Technol*.** 75, 55–61. (10.1016/j.compscitech.2012.12.006)

[24] Yu B, Bradley RS, Soutis C, Hogg PJ, Withers PJ 2015 2D and 3D imaging of fatigue failure mechanisms of 3D woven composites. *Compos. A, Appl. S* 77, 37–49. (10.1016/j.compositesa.2015.06.013)

[25] ASTM D3039/D 3039M. 1995 *Standard test method for tensile properties of polymer matrix composite materials*. West Conshohocken, PA: ASTM International. See http://www.astm.org.

[26] Bull DJ, Sinclair I, Spearing SM 2013 Partial volume correction for approximating crack opening displacements in CFRP material obtained from micro-focus X-ray CT scans. *Compos. Sci. Technol*.** 81, 9–16. (10.1016/j.compscitech.2013.03.017)

